# Development and validation of a machine learning-based predictive model for early outcomes following combined suction-assisted lipectomy and lymphovenous anastomosis in breast cancer-related lymphedema: a retrospective cohort study

**DOI:** 10.3389/fonc.2026.1828402

**Published:** 2026-05-07

**Authors:** Yonghao Cui, Hao Dong, Zixuan Yao, Shuai Pang, Yuguang Sun, Song Xia, Wenbin Shen

**Affiliations:** Department of Lymphatic Surgery, Capital Medical University Affiliated Beijing Shijitan Hospital; Clinical Center for Lymphatic Disorders, CMU, Beijing, China

**Keywords:** breast cancer-related lymphedema, lymphovenous anastomosis, machine learning, predictive model, suction-assisted lipectomy, support vector machine

## Abstract

**Background:**

Breast cancer-related lymphedema (BCRL) significantly compromises quality of life. Although combined suction-assisted lipectomy (SAL) and lymphovenous anastomosis (LVA) is effective, outcomes vary considerably among patients. Currently, tools for early postoperative risk stratification are lacking.

**Methods:**

We retrospectively reviewed data from BCRL patients who underwent combined SAL and LVA at Beijing Shijitan Hospitalfrom June 2018 to June 2025. Predictive variables were selected using the Least Absolute Shrinkage and Selection Operator (LASSO) regression combined with bootstrap resampling (B = 1,000). Seven algorithms—including logistic regression (LR), decision tree (DT), random forest (RF), eXtreme Gradient Boosting (XGBoost), Light Gradient Boosting Machine (LightGBM), support vector machine (SVM), and artificial neural network (ANN) were compared. Model performance was evaluated using the area under the receiver operating characteristic curve (AUC), calibration plots, decision curve analysis (DCA), and Brier score. SHapley Additive exPlanations (SHAP) analysis was conducted for model interpretation, and a web-based prediction tool was developed.

**Results:**

A total of 300 patients were enrolled (training set: n=211; validation set: n=89). The rate of satisfactory outcomes at 6 months was 72.3%. LASSO and bootstrap validation identified three stable predictors: postoperative excess limb volume (selection frequency: 100%), disease duration (83.6%), and disease severity grade (83.4%). Among the seven models, SVM exhibited the optimal balance of discrimination and clinical utility in the validation set: AUC 0.891 (95% CI: 0.812–0.970), sensitivity 90.8%, specificity 62.5%, F1-score 0.887, and Brier score 0.119. DCA indicated net clinical benefit within the threshold range of 0.1–0.6. Although ANN achieved a higher AUC than SVM (0.903 vs. 0.891) (DeLong test, P = 0.532), SVM demonstrated superior sensitivity (90.8% vs. 89.2%), F1-score (0.887 vs. 0.879), and Cohen’s kappa (0.555 vs. 0.531).Furthermore, SVM’s structural risk minimization principle conferred superior generalization stability compared with ANN’s empirical risk minimization, making it more suitable for small-sample clinical settings. SHAP analysis revealed that postoperative excess volume was the strongest predictor.

**Conclusion:**

The 3-variable SVM model effectively predicts 6-month outcomes following combined surgery for BCRL. Integrated with SHAP analysis and a web-based tool, this model enables early postoperative risk stratification to identify high-risk patients requiring closer monitoring, providing a reference for future standardized rehabilitation protocols.

## 1 Introduction

Lymphedema is a chronic, progressive disease characterized by the accumulation of protein-rich interstitial fluid and fibroadipose tissue in subcutaneous layers secondary to lymphatic drainage impairment (1). Breast cancer-related lymphedema (BCRL) represents one of the most common and long-term complications following breast cancer treatment, with a cumulative incidence of 33% at 6–18 months postoperatively (2). As survival rates for breast cancer patients have improved, the number of patients developing BCRL has increased correspondingly. The resulting limb deformity, functional impairment, and recurrent cellulitis impose substantial physical, psychological, and economic burdens on patients (3, 4). Current treatments for BCRL include conservative and surgical interventions. Conservative therapy, primarily complex decongestive therapy (CDT) combined with compression, represents the first-line approach but is associated with complex protocols, poor patient compliance, and limited efficacy in advanced fibrotic stages (5, 6).

For advanced lymphedema, surgical intervention offers superior alternatives. Surgical approaches include debulking procedures and physiological reconstruction. Among debulking techniques, suction-assisted lipectomy (SAL) was initially proposed by O’Brien (7) and refined by Brorson (8). This technique improves limb contour by removing hypertrophic adipose tissue and fibrotic deposits. In a study by Damstra et al. (9), the mean excess volume decreased from 1.47 preoperatively to 1.09 postoperatively, further reducing to 0.96 at 12 months. Similarly, Schaverien et al. (10) reported that patients maintained a mean volume ratio of 1.09 at 5 years, with concomitant improvements in anxiety and depression symptoms. Boyages et al. (11) reported a reduction in mean excess volume from 45.1% to 13.2%. Although SAL effectively reduces limb volume, it fails to address lymphatic dysfunction, leaving patients at risk for recurrence. Physiological reconstruction includes lymphaticovenous anastomosis (LVA) and vascularized lymph node transfer (VLNT), with LVA being a commonly used technique. LVA restores lymphatic drainage by anastomosing lymphatic vessels to subcutaneous veins (12, 13). In a prospective series of 100 patients, Chang DW et al. (14) reported limb volume reductions of 33%, 36%, and 42% at 3, 6, and 12 months postoperatively, respectively. While LVA resolves lymphatic congestion and may allow discontinuation of compression garments in selected patients, its impact on limb volume reduction remains modest, and it cannot effectively address established fibrosis. Single-modality approaches exhibit distinct limitations: SAL reduces volume but fails to reconstruct lymphatic pathways, whereas LVA alone provides limited benefit for patients with severe fibrosis and adipose hypertrophy (15).

Our institution employs a sequential “debulking-first, reconstruction-second” strategy, performing SAL followed by deep LVA 2–4 months later, supplemented with postoperative compression therapy. Utilizing deep lymphatic vessels for anastomosis circumvents the destruction of superficial lymphatics caused by liposuction, enabling both volume reduction and long-term lymphatic drainage restoration. This combined approach has demonstrated superior outcomes compared to single-modality treatments, with the median excess volume decreasing from 50.7% to 0.6% postoperatively, along with a reduced incidence of postoperative erysipelas (16).

However, we observed that some patients failed to achieve optimal outcomes at 6-month follow-up, resulting in significant psychological distress. This may be attributed to uniform postoperative rehabilitation protocols without individualized treatment strategies. Therefore, early identification and timely adjustment of therapeutic approaches are crucial to prevent residual swelling. However, tools for early postoperative identification of high-risk patients are currently unavailable.

Machine learning has been increasingly applied in constructing clinical risk prediction models, providing researchers with powerful statistical methods to capture associations between patient characteristics and outcomes while enabling objective data integration for clinical prognosis prediction (17, 18). This study aims to construct and validate a machine learning model for predicting 6-month postoperative outcomes in BCRL patients undergoing combined surgeries, based on indicators available at patient discharge. Building upon established efficacy of combined SAL-LVA (16, 19, 20) and predictive modeling applications in lymphedema, this study specifically addresses early postoperative risk stratification—utilizing discharge-available indicators to forecast 6-month outcomes. By focusing on the postoperative day 7 window, we developed a practical tool for identifying high-risk patients requiring closer monitoring, enabling timely risk assessment at discharge. We compared logistic regression and six machine learning algorithms to develop this interpretable predictive tool.

## 2 Materials and methods

### 2.1 Study design and participants

**Figure 1:**
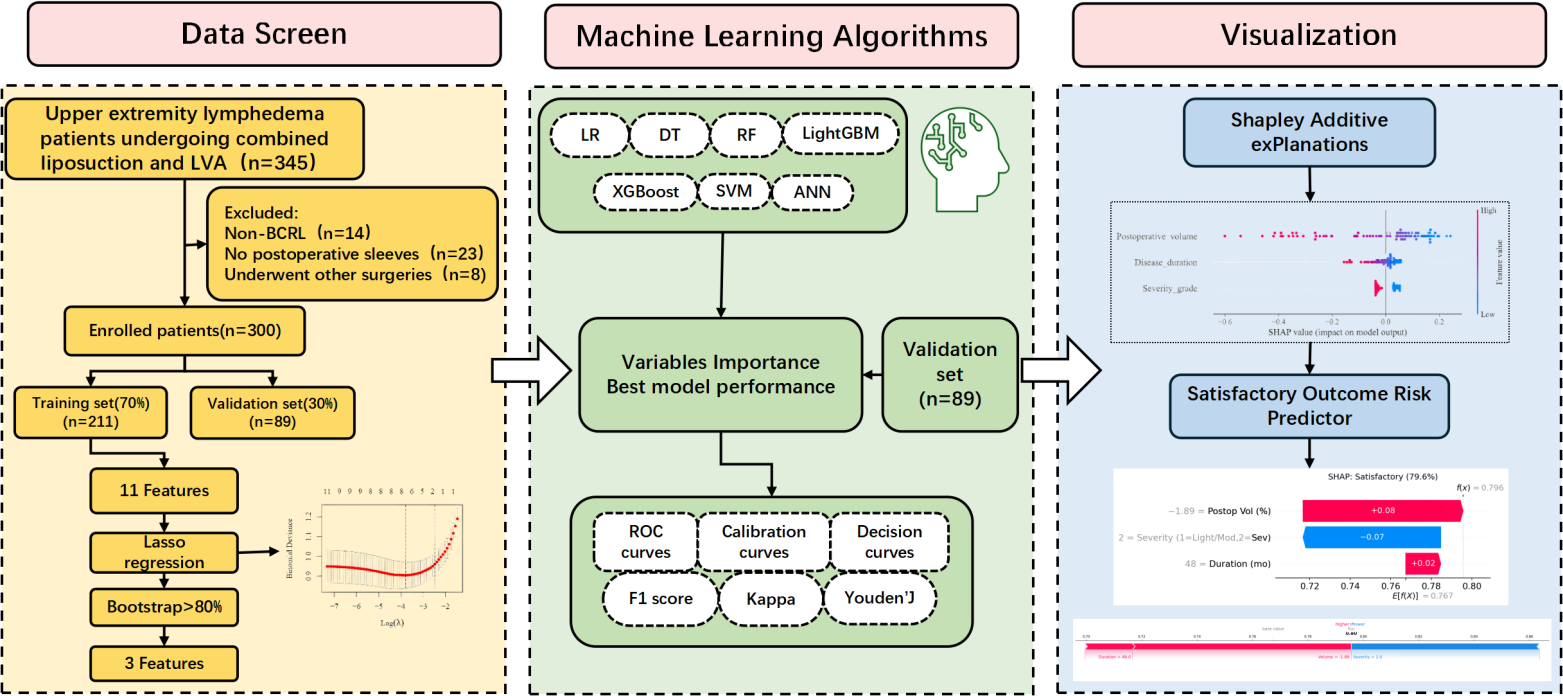
The Overall flowchart of the study.

This retrospective cohort study included 345 upper extremity lymphedema patients who underwent combined SAL and LVA at our institution between June 2018 and June 2025. Inclusion criteria were: (1) clinically diagnosed unilateral secondary lymphedema following breast cancer surgery; (2) adherence to standardized postoperative compression therapy; (3) International Society of Lymphology (ISL) stage II or higher. Exclusion criteria included: non-BCRL lymphedema (n=14), non-adherence to postoperative compression therapy (n=23), and underwent other upper extremity surgery (n=8) (**Figure 1**). All 300 included patients had complete data for all 11 candidate variables and the 3 final predictors; no missing data imputation was required. Data completeness was ensured by the retrospective chart review process, with all measurements (baseline, intraoperative, and postoperative day 7) available for every included case.

### 2.2 Definitions and outcomes

The primary endpoint was treatment outcome at 6 months, categorized as either satisfactory outcome (bilateral limb volume difference<5%) or residual swelling (≥5%). This threshold was selected based on three considerations: firstly, alignment with ISL 2023 consensus defining the lower limit of mild lymphedema as >5% excess (21); secondly, our institutional emphasis on complete volume normalization rather than partial improvement; thirdly, adoption of a conservative threshold that ensures observed volume reductions represent clinically meaningful improvement rather than fluctuations inherent in circumferential volumetric assessment (22). We acknowledge that alternative thresholds (e.g.,<10%) are employed in other studies to define meaningful clinical improvement; however, our analysis specifically targeted prediction of complete resolution below the ISL pathological threshold.

### 2.3 Data collection

We collected the following 11 variables: (1) Demographics: age, body mass index (BMI, kg/m²); (2) Disease characteristics: lymphedema duration (months), preoperative limb volume excess percentage, disease severity grade (based on ISL criteria and preoperative volume excess: mild 5–20%, moderate 20–40%, severe >40%; dichotomized as mild-moderate vs. severe) (19), hand swelling (present/absent, defined as circumference difference >2 cm) (23), history of erysipelas, history of chemotherapy/radiotherapy; (3) Intraoperative variables: liposuction volume (mL), number of lymphatic anastomoses; (4) Postoperative measurement: postoperative excess volume at day 7.

Bilateral volume excess was calculated using circumferential measurements at 11 anatomical landmarks using the Frustum Formula: $V=\frac{\left(C_{1}^{2}+C_{2}^{2}+C_{1}C_{2}\right)h}{12\pi }$ (22). The percentage excess was calculated as: $\frac{V_{affected}-V_{healthy}}{V_{healthy}}\times 100\%$. All measurements were performed by two trained rehabilitation therapists; remeasurement was required for discrepancies >5%.

### 2.4 Variable selection

Using random stratified sampling at a 7:3 ratio, the dataset was divided into training (n=211) and validation (n=89) sets, ensuring comparable outcome distributions between groups. Variable selection was performed using the Least Absolute Shrinkage and Selection Operator (LASSO) regression with 10-fold cross-validation to determine the optimal regularization parameter (lambda.min). Subsequently, bootstrap resampling (B = 1,000) was performed to assess selection stability, retaining variables with selection frequency >80% for model development (24).

### 2.5 Model development and evaluation

Seven algorithms were compared: (1) logistic regression (LR); (2) decision tree (DT); (3) random forest (RF); (4) eXtreme Gradient Boosting (XGBoost); (5) Light Gradient Boosting Machine (LightGBM); (6) support vector machine (SVM); and (7) artificial neural network (ANN). Hyperparameter tuning was performed using Grid Search combined with 5-fold cross-validation to optimize model performance. Model performance was evaluated using the area under the receiver operating characteristic curve (AUC), calibration plots, decision curve analysis (DCA), and Brier score. Discrimination was assessed using sensitivity, specificity, F1-score, Cohen’s kappa coefficient, and Youden index. To address the limitation of single random split, bootstrap optimism correction (B = 1,000) was performed on the full cohort (n=300) by resampling with replacement and calculating the mean optimism (bootstrap performance minus apparent performance). The optimism-corrected AUC was reported to provide a robust estimate of model generalizability. Following model selection, SHAP (SHapley Additive exPlanations) analysis was performed for model interpretation (25), and a web-based prediction tool was developed using Gradio.

### 2.6 Statistical analysis

Statistical analyses were performed using R version 4.5.1 and Python version 3.10.4. Continuous variables are presented as mean ± standard deviation or median [interquartile range] and were compared using independent t-tests or Mann-Whitney U tests. Categorical variables are presented as frequency (percentage) and were compared using Chi-square or Fisher’s exact tests. P-value<0.05 was considered statistically significant.

## 3 Results

### 3.1 Baseline characteristics

A total of 300 patients were included (training set: n=211 [70.3%]; validation set: n=89 [29.7%]). The cohort comprised middle-aged to elderly women with a median age of 57.0 years [50.0, 64.0] and a median BMI of 25.7 kg/m² [23.4, 28.4]. Approximately 75% were overweight or obese. The median disease duration was 48 months [24, 96]. Overall, 77% had received combined chemoradiotherapy, 22% had a history of erysipelas, and 47.3% exhibited hand swelling. Regarding severity, 45% were mild-to-moderate and 55% had severe lymphedema. The median preoperative excess volume was 42.5% [27.8, 56.7%]. The median liposuction volume was 1,200 mL [1,000, 1,500], and the median number of lymphatic anastomoses was 5.0 [4.0, 6.0]. The median postoperative excess volume was -0.41% [-6.98, 8.50%], representing a 42.9% reduction from baseline. The overall rate of satisfactory outcomes was 72.3%(217/300), while 27.7% had residual swelling. Between-group comparisons revealed that patients with residual swelling were significantly older, had longer disease duration, higher prevalence of hand swelling, greater proportion of severe lymphedema, larger preoperative excess volume, and higher postoperative excess volume (all P<0.05) (**Table 1**). No significant differences were observed between training and validation sets in terms of age, BMI, disease duration, liposuction volume, number of anastomoses, preoperative excess volume, postoperative excess volume, chemoradiotherapy history, erysipelas history, hand swelling, or severity (all P>0.05) (**Table 2**).

**Table 1:** Comparison of baseline data between residual swell group and satisfactory outcome group.

Variables	Total(n=300)	Residual swell(n=83)	Satisfactory outcome(n=217)	*P*
Chemoradiotherapy (%)				
none	5 (1.67)	2 (2.41)	3 (1.38)	
either	64 (21.33)	12 (14.46)	52 (23.96)	0.175
both	231 (77.00)	69 (83.13)	162 (74.65)	
Erysipelas (%)	66 (22.00)	23 (27.71)	43 (19.82)	0.187
Hand swell (%)	142 (47.33)	56 (67.47)	86 (39.63)	<0.001
Severity grade (%)				
mild and moderate	135 (45.00)	14 (16.87)	121 (55.76)	<0.001
severe	165 (55.00)	69 (83.13)	96 (44.24)	
Age (median [IQR])	57.00 [50.00, 64.00]	60.00 [53.50, 67.00]	56.00 [50.00, 63.00]	0.006
BMI (median [IQR])	25.66 [23.42, 28.43]	25.71 [23.17, 27.87]	25.59 [23.43, 28.44]	0.896
Disease duration (median [IQR])	48.00 [24.00, 96.00]	60.00 [24.00, 114.00]	48.00 [18.00, 72.00]	0.004
Liposuction volume (median [IQR])	1200.00 [1000.00, 1500.00]	1300.00 [1000.00, 1500.00]	1200.00 [950.00, 1500.00]	0.125
Anastomosis number (median [IQR])	5.00 [4.00, 6.00]	5.00 [4.00, 6.00]	5.00 [4.00, 6.00]	0.234
Preoperative volume (median [IQR])	42.52 [27.81, 56.68]	56.69 [45.25, 72.93]	35.74 [24.38, 48.85]	<0.001
Postoperative volume (median [IQR])	-0.41 [-6.98, 8.50]	11.10 [4.01, 17.18]	-3.35 [-10.45, 1.90]	<0.001

IQR, Interquartile range.

**Table 2:** Training set and validation set variability analysis.

Variables	Total (n = 300)	Validation (n = 89)	Training (n = 211)	*P*
Chemoradiotherapy(%)				
none	5 (1.67)	0 (0.00)	5 (2.37)	0.18
either	64 (21.33)	23 (25.84)	41 (19.43)	
both	231 (77.00)	66 (74.16)	165 (78.20)	
Erysipelas(%)	66 (22.00)	21 (23.60)	45 (21.33)	0.66
Hand swell(%)	142 (47.33)	39 (43.82)	103 (48.82)	0.43
Severity grade(%)				
mild and moderate	135 (45.00)	42 (47.19)	93 (44.08)	0.62
severe	165 (55.00)	47 (52.81)	118 (55.92)	
Age (median [IQR])	57.00 [50.00, 64.00]	57.00 [52.00, 64.00]	56.00 [50.00, 64.50]	0.55
BMI (median [IQR])	25.66 [23.42, 28.43]	25.64 [22.96, 29.02]	25.71 [23.55, 28.13]	0.78
Disease duration (median [IQR])	48.00 [24.00, 96.00]	60.00 [24.00, 96.00]	48.00 [21.00, 84.00]	0.39
Liposuction volume (median [IQR])	1200.00 [1000.00, 1500.00]	1300.00 [1000.00, 1500.00]	1200.00 [1000.00, 1600.00]	1.00
Anastomosis number (median [IQR])	5.00 [4.00, 6.00]	5.00 [4.00, 6.00]	5.00 [4.00, 6.00]	0.40
Preoperative volume (median [IQR])	42.52 [27.81, 56.68]	42.08 [27.49, 54.58]	43.08 [28.02, 56.81]	0.66
Postoperative volume (median [IQR])	-0.41 [-6.98, 8.50]	-0.06 [-5.75, 9.20]	-0.47 [-7.43, 7.80]	0.22

### 3.2 Variable selection

**Figure 2:**
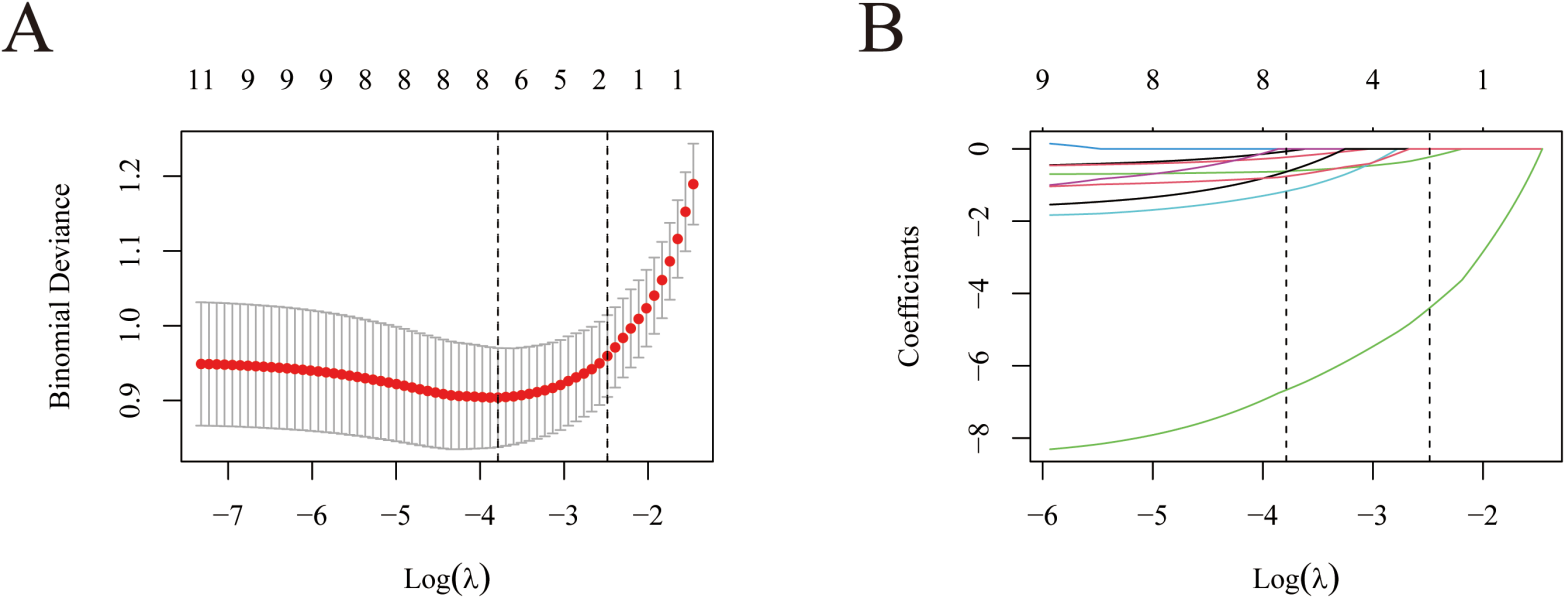
Presentation of the results of the LASSO regression analysis. **(A)** LASSO regression model factor selection: left dashed line represents the optional lambda value (lambda.min), while the right dashed line marks the lambda value within one standard error of the optimal (lambda.1se); **(B)** LASSO regression model screening variable trajectories.

LASSO regression with 10-fold cross-validation selected 7 variables from 11 candidates (**Figures 2A, B**). Bootstrap validation (B = 1,000) further identified three stable predictors: postoperative excess volume (100%), disease duration (83.6%), and severity grade (83.4%) (**Table 3**). Notably, preoperative volume excess and severity grade exhibited substantial collinearity (Pearson r = 0.672, P< 0.001). LASSO regression inherently handles such correlated predictors via L1 regularization. Bootstrap validation (B = 1,000) demonstrated superior selection stability for severity grade (83.4%) compared to preoperative volume (61.3%), supporting retention of the categorized variable despite their correlation.

**Table 3:** Bootstrap stability assessment of LASSO variable selection.

Characteristics	Frequency
Postoperative volume	100.0
Disease duration	83.6
Severity grade	83.4
Anastomosis number	74.6
Hand swell	68.7
Preoperative volume	61.3
Erysipelas	58
Liposuction volume	48.6
Age	38.2
Chemoradiotherapy	37.8
BMI	33.3

### 3.3 Model performance

#### 3.3.1 Training set performance

In the training set, LightGBM achieved the highest AUC (0.986), accuracy (0.943), F1-score (0.960), and Cohen’s kappa (0.860). RF demonstrated perfect discrimination (AUC = 1.000), suggesting overfitting (**Table 4**).

**Table 4:** Detailed performance metrics of various machine learning models for predicting satisfactory outcome in BCRL patients across training set.

Model	AUC	Accuracy	Precision	Sensitivity	Specificity	F1 Score	Kappa	Youden’s J	PPV	NPV
LR	0.853	0.801	0.831	0.908	0.525	0.868	0.467	0.433	0.831	0.689
DT	0.918	0.853	0.917	0.875	0.797	0.896	0.648	0.672	0.917	0.712
RF	1.000	1.000	1.000	1.000	1.000	1.000	1.000	1.000	1.000	1.000
XGB	0.943	0.858	0.859	0.961	0.593	0.907	0.611	0.554	0.859	0.854
LGBM	0.986	0.943	0.967	0.954	0.915	0.960	0.860	0.869	0.967	0.885
SVM	0.854	0.787	0.802	0.934	0.407	0.863	0.392	0.341	0.802	0.706
ANN	0.853	0.801	0.816	0.934	0.458	0.871	0.442	0.392	0.816	0.730

**Figure 3:**
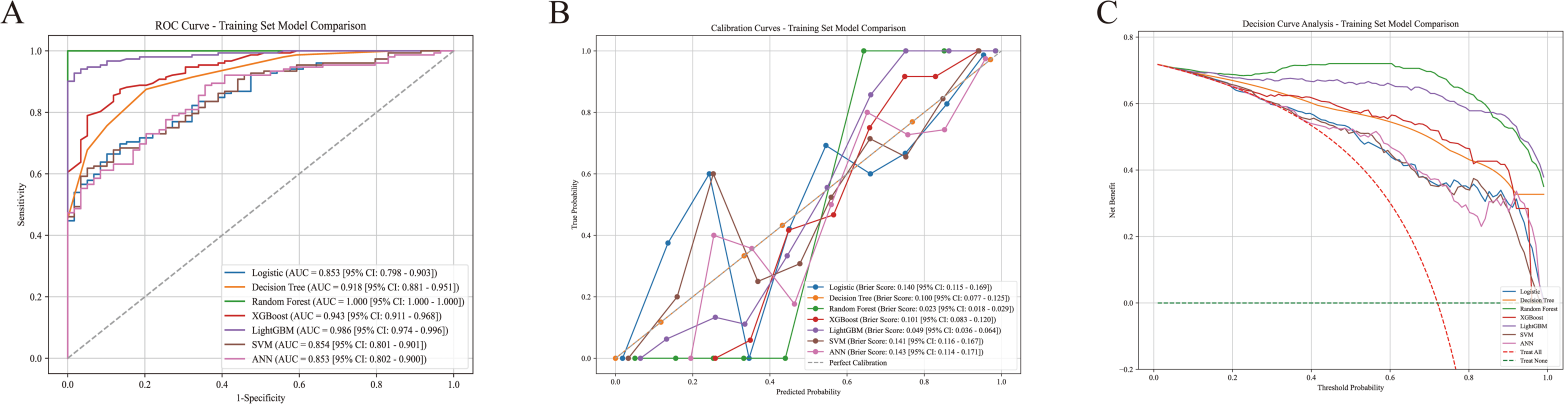
The performance and comparison of seven different predictive models for the training set. **(A)** Receiver operating characteristic (ROC) curves: the ordinate represents sensitivity, and the abscissa represents 1-specificity. AUC: area under the curve, 95%CI: 95% confidence interval; **(B)** Calibration curves: The ordinate represents the actual event occurrence probability, the abscissa represents the model predicted probability, and the dashed line is the perfect calibration line; **(C)** Decision curve analysis (DCA): the ordinate represents the net benefit, the abscissa represents the clinical decision threshold probability, “treat all” is the reference curve for treating all research subjects, and “treat none” is the reference curve for not treating any research subjects.

ROC analysis (**Figure 3A**) showed that tree models outperformed traditional models; RF achieved perfect discrimination (AUC = 1.000, 95% CI: 1.000–1.000), followed by LightGBM (0.986), XGBoost (0.943), and DT (0.918). LR, SVM, and ANN showed comparable discrimination (AUC 0.853–0.854).

Calibration plots (**Figure 3B**) showed that all models approximated the ideal diagonal. The Brier scores indicated optimal calibration for RF (0.023) and LightGBM (0.049), with reasonable calibration for LR, SVM, and ANN (0.140–0.143).

DCA (**Figure 3C**) demonstrated that all models provided positive net benefit across threshold probabilities of 0.0–1.0.

#### 3.3.2 Validation set performance

**Figure 4:**
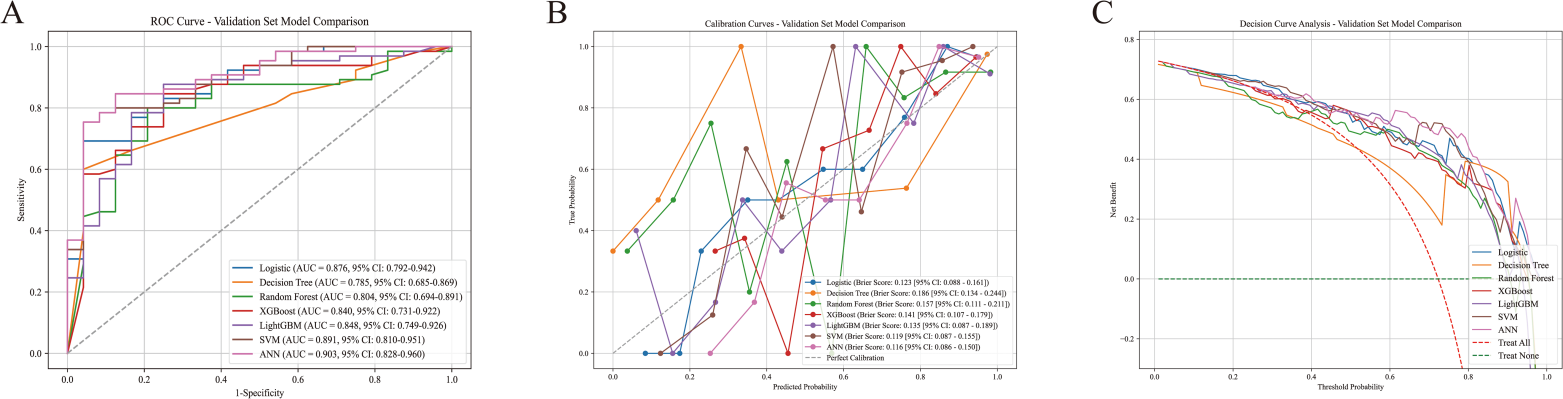
The performance and comparison of seven different predictive models for the validation set. **(A)** ROC curves; **(B)** calibration curve; **(C)** Decision curve analysis.

In the validation set (**Table 5**, **Figures 4A–C**), SVM demonstrated optimal comprehensive performance: AUC 0.891 (95% CI: 0.812–0.970), sensitivity 90.8%, specificity 62.5%, F1-score 0.887, Cohen’s kappa 0.555, and Youden index 0.533. ANN achieved the highest AUC (0.903) but lower F1 (0.879) and kappa (0.531) compared with SVM. LR performed robustly (AUC 0.876, sensitivity 90.8%). Tree models showed inconsistent performance: LightGBM performed reasonably (AUC 0.848, F1 0.884, kappa 0.578), but XGBoost had low specificity (0.542), RF showed moderate kappa (0.438), and DT performed poorly (kappa 0.277).

**Table 5:** Detailed performance metrics of various machine learning models for predicting satisfactory outcome in BCRL patients across validation set.

Model	AUC	Accuracy	Precision	Sensitivity	Specificity	F1 Score	Kappa	Youden’s J	PPV	NPV
LR	0.876	0.820	0.855	0.908	0.583	0.881	0.518	0.491	0.855	0.700
DT	0.785	0.719	0.803	0.815	0.458	0.809	0.277	0.274	0.803	0.478
RF	0.804	0.764	0.867	0.800	0.667	0.832	0.438	0.467	0.867	0.552
XGB	0.840	0.831	0.847	0.938	0.542	0.891	0.529	0.480	0.847	0.765
LGBM	0.848	0.831	0.891	0.877	0.708	0.884	0.578	0.585	0.891	0.680
SVM	0.891	0.831	0.868	0.908	0.625	0.887	0.555	0.533	0.868	0.714
ANN	0.903	0.820	0.866	0.892	0.625	0.879	0.531	0.517	0.866	0.682

ROC analysis (**Figure 4A**) showed that SVM, ANN, and LR clustered in the upper left quadrant (AUC>0.87). DeLong tests confirmed no statistically significant differences among these models (all P > 0.05) (**Table 6**).

**Table 6:** The AUC DeLong test results.

Model1	Model2	AUC1	AUC2	P
SVM	ANN	0.891	0.903	0.532
SVM	LR	0.891	0.876	0.435
ANN	LR	0.903	0.876	0.212

Calibration plots (**Figure 4B**) showed marked divergence in the validation set. Tree models demonstrated poor calibration (Brier score: RF 0.157, DT 0.186). Traditional models maintained stable calibration (Brier score: ANN 0.116, SVM 0.119, LR 0.123).

DCA (**Figure 4C**) showed that SVM, ANN, and LR maintained high net benefit, significantly outperforming tree models.

#### 3.3.3 Optimal model selection

Feature scaling was uniformly omitted across all algorithms to ensure methodological comparability. This approach was justified because: (1) linear models (SVM with linear kernel and LR) inherently adjust weight coefficients to accommodate heterogeneous feature scales; (2) tree-based models (DT, RF, XGB, LGBM) rely on rank-order splitting and are scale-invariant; (3) the single-layer ANN with logistic activation tolerated unscaled inputs in this low-dimensional setting (p=3) through hyperparameter optimization. Thus, preprocessing uniformity was maintained across all seven algorithms. **Table 7** presents the final optimal hyperparameters selected for each algorithm via Grid Search with 5-fold cross-validation.

**Table 7:** Optimal parameters for each model.

Full super-parameters of techniques
Decision Tree(DT):
DT Classifer: {‘ccp_alpha’: 0.0, ‘max_depth’: 5, ‘max_features’: ‘sqrt’, ‘min_samples_split’: 20}
Random Forest(RF):
RF Classifer:n_estimators = 250, max_features = 2
eXtreme Gradient Boosting(XGBoost):
XGBoost Classifer: {‘learning_rate’: 0.01, ‘max_depth’: 3, ‘n_estimators’: 200, ‘subsample’: 1.0}
Light Gradient Boosting Machine(LightGBM):
LightGBM Classifer: {‘colsample_bytree’: 1.0, ‘learning_rate’: 0.1, ‘n_estimators’: 100, ‘num_leaves’: 31, ‘subsample’: 0.6}
Support Vector Machine(SVM):
SVM Classifer: {‘C’: 0.1, ‘degree’: 2, ‘gamma’: ‘scale’, ‘kernel’: ‘linear’}
Artificial Neural Network(ANN):
ANN Classifer: {‘activation’: ‘logistic’, ‘hidden_layer_sizes’: (100)},

Although ANN achieved a marginally higher AUC than SVM (0.903 vs. 0.891), this difference was not statistically significant (DeLong test, P>0.05). However, SVM demonstrated superior clinical performance with higher sensitivity (90.8% vs. 89.2%), F1-score (0.887 vs. 0.879), and Cohen’s kappa (0.555 vs. 0.531). Furthermore, SVM’s structural risk minimization principle confers better generalization stability compared with ANN’s empirical risk minimization. Therefore, SVM was selected as the optimal predictive model.

This generalization advantage was confirmed by bootstrap optimism correction (B = 1,000) on the full cohort (n=300), which yielded an optimism-corrected AUC of 0.863 (mean optimism: -0.000) for the SVM model, indicating minimal overfitting. With 65 events (satisfactory outcomes) in the training set and 3 final predictors, the events-per-variable (EPV) ratio was 21.7, exceeding the recommended minimum of 10 for stable logistic regression estimates. This estimate is consistent with the validation set performance (AUC 0.891), supporting robust model stability despite the modest validation sample size (n=89).

#### 3.3.4 Sensitivity analysis: impact of excluding postoperative excess volume

To address potential circularity concerns, we performed sensitivity analysis excluding postoperative excess volume. While the SVM algorithm showed limited discrimination with only disease duration and severity grade (AUC 0.374), the LightGBM model achieved the best performance among reduced models (AUC 0.726, 95% CI: 0.595–0.831). (**Supplementary Table 1**) DeLong test confirmed significant performance degradation (AUC 0.891 vs. 0.726, Z = 2.04, P = 0.041), indicating that postoperative excess volume contributes unique prognostic information beyond the other two variables. Notably, despite excluding the strongest predictor, the remaining model still demonstrated substantial discrimination (AUC 0.726 > 0.5), confirming that disease duration and severity grade retain independent predictive value.

### 3.4 Model interpretability and application

**Figure 5:**
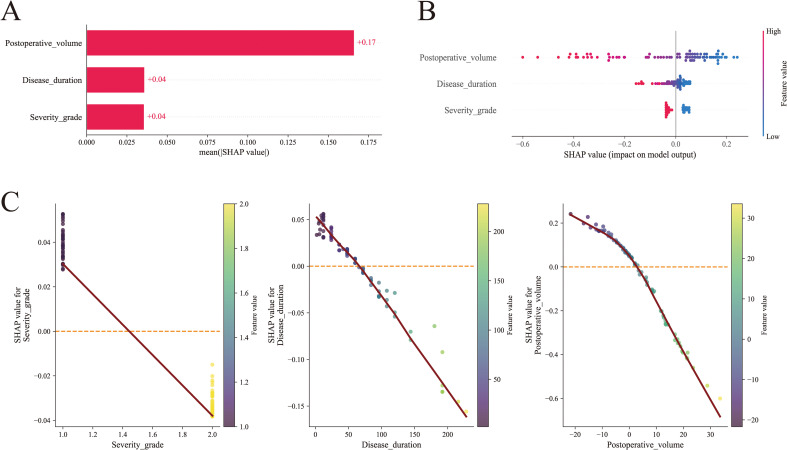
SHAP global interpretability analysis of the SVM model. **(A)** Bar plot: SHAP value of risk factors of all participants in bar plot. Variables were sequenced according to the mean SHAP value in SVM. High SHAP value represents greater impact on the happening of satisfactory outcome **(B)** Summary plot: SHAP value of risk factors of all participants in summary plot. The color of the spot represented the value of this feature. **(C)** Dependence plot: The relationship between the values of features and their corresponding SHAP values. The abscissa represents the feature value, the ordinate represents the SHAP value, and the red curve is the locally weighted regression line, reflecting the overall trend between the feature and SHAP values;.

**Figure 6:**
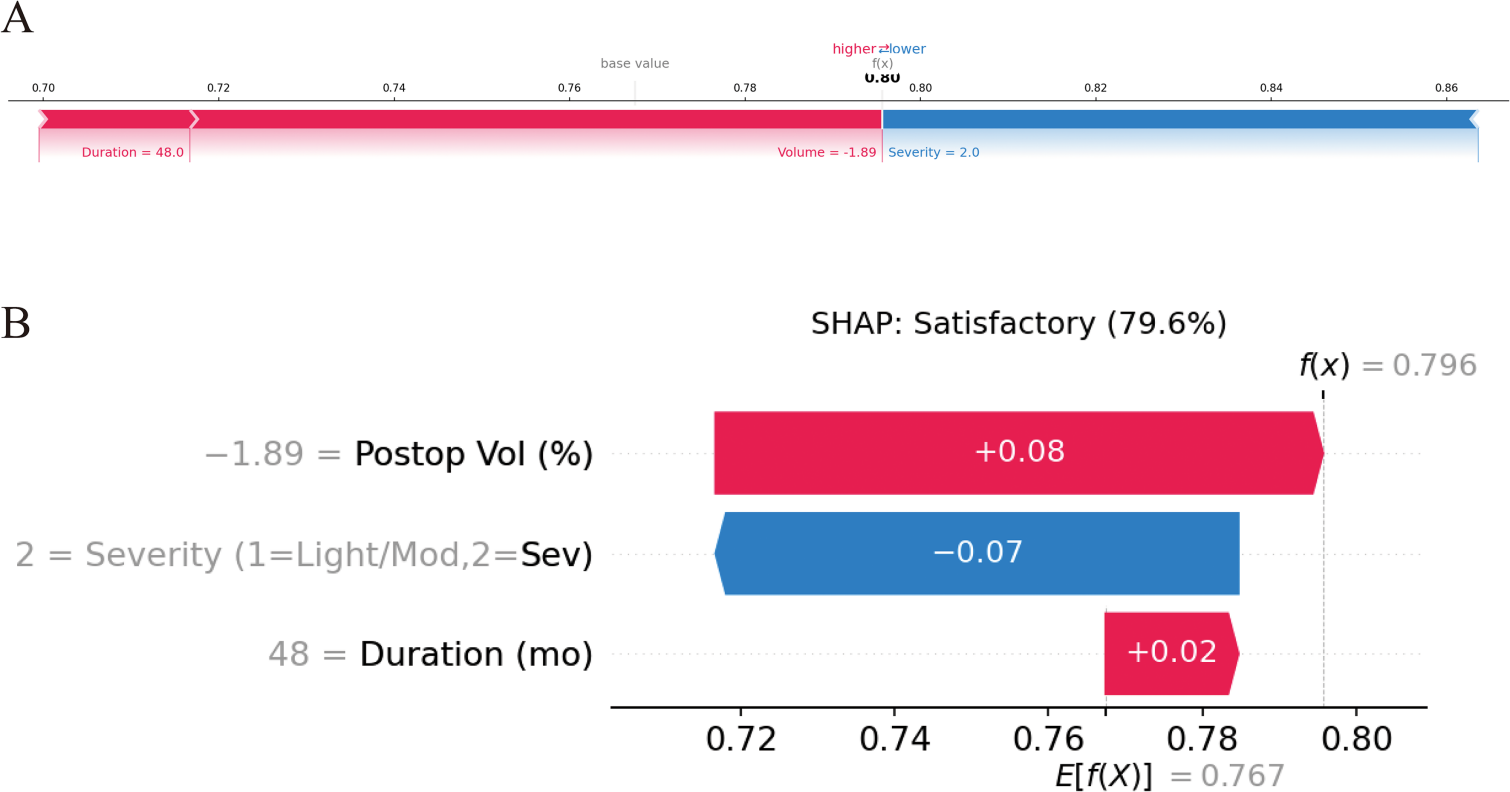
SHAP individual prediction explanations for the SVM model. **(A)** Force plot: red colour represented a positive influence of the feature on satisfactory outcome. Blue colour represented the opposite. The final value of this individual was 0.796, greater than the base value (0.767), which indicated the probability of satisfactory outcome; **(B)** Waterfall plot: It was an extended form of force plot.

**Figures 5A–C** present SHAP plots, which provide interpretability for the SVM model predictions. **Figure 5A** displays the ranked importance of variables in the model: postoperative excess volume, disease duration and severity grade. **Figure 5B** provides insights into the SVM model predictions, illustrating each feature’s impact on the outcome variable. The x-axis represents SHAP values, where higher values indicate a stronger positive influence on treatment satisfaction (increasing the likelihood of a satisfactory outcome). Postoperative excess volume has the greatest impact on the outcome, followed by duration of lymphedema and disease severity. The color gradient from red to blue indicates feature magnitude, with red representing high values and blue low values. Higher values for these three variables correspond to smaller SHAP values, indicating a greater likelihood of residual swelling and a negative correlation with treatment satisfaction. This aligns with the negative correlations shown in **Figure 5C**. This study utilized Gradio on the deployable web platform Hugging Face to visualize and implement the predictive model for basic applications (https://huggingface.co/spaces/hhhhhhyyyyyy/Postoperative_6_month_efficacy_of_BCRL_treated_with_liposuction_combined_with_LVA). This provides an online tool for assessing the six-month postoperative efficacy of combined surgical treatment for BCRL patients. By directly entering clinical data into the designated text fields on the webpage, users can easily obtain the desired predictive results. **Figures 6A, B** respectively display the interpretability analysis for the same sample.

## 4 Discussion

With improved survival rates for breast cancer patients, BCRL has become a major complication affecting long-term quality of life (2). Combined SAL-LVA has demonstrated efficacy in advanced BCRL (16, 19, 20). While predictive models for lymphedema remain limited, particularly for postoperative outcomes following combined surgical treatment, this study developed and validated a model to predict 6-month efficacy after SAL-LVA using discharge-available indicators (postoperative day 7). This provides a practical tool for early risk stratification and closer monitoring of high-risk patients.

The pathophysiology of lymphedema follows a “stasis-inflammation-fibrosis” cycle (26). Lymphatic drainage impairment leads to interstitial fluid accumulation, triggering persistent inflammatory responses that drive adipose tissue deposition and fibrosis. While SAL effectively reduces limb volume, it fails to restore lymphatic drainage, predisposing patients to recurrence; LVA alone offers limited benefit for patients with severe fibrosis and adipose tissue hypertrophy. Our combined approach utilizing deep lymphatic vessel anastomosis following SAL addresses both volume reduction and long-term lymphatic drainage restoration (27), distinguishing it from traditional superficial LVA approaches (16). This strategy enables lymphatic reconstruction even in limbs that have undergone liposuction, which would otherwise preclude superficial LVA. Furthermore, other studies employing LVA combined with liposuction have also achieved favorable outcomes, confirming the feasibility and efficacy of integrating these two modalities (19, 20).

This study identified three independent predictors through LASSO regression combined with Bootstrap validation: postoperative excess volume, disease duration, and severity grade. These variables capture prognostic information across three dimensions: immediate anatomical improvement, temporal pathological progression, and cumulative anatomical burden.

Postoperative excess limb volume at day 7 reflects the immediate anatomical improvement following surgery. Currently, SAL has completed volume reduction, and the residual excess primarily reflects the initial efficacy of lymphatic reconstruction. Longitudinal studies of postoperative volume changes indicate that early volume improvement serves as a sensitive predictor of long-term outcomes. Demmer et al. (28) observed that in patients undergoing microsurgical lymphatic vessel transplantation, the relative volume difference decreased significantly from 24.3% preoperatively to 13.8% during the early postoperative period (P = 0.05), and this early improvement correlated significantly with outcomes at 7.8 months (P<0.01). This finding suggests that early postoperative volume parameters can predict long-term efficacy. Moreover, postoperative day 7 coincides with the critical endothelial healing window. If effective lymphovenous shunting is established by this time, the lymphatic load in the affected limb decreases immediately, and the volume excess should serve as the baseline floor for long-term recovery. Conversely, if the residual volume excess remains substantial at postoperative day 7, this suggests poor anastomotic function or severe lymphatic stasis, indicating high risk for residual swelling at long-term follow-up.

Disease duration reflects the temporal pathological progression of lymphedema. As duration extends, tissues transition from reversible edema to irreversible fibrosis, directly impacting the synergistic efficacy of combined surgery. Research has demonstrated that as disease progresses, lymphatic vessel structures become destroyed, and even with established anastomoses, lymphatic drainage remains difficult to restore; therefore, patients with shorter disease duration are more likely to achieve complete relief through surgical intervention (29). Additionally, in studies of secondary lower extremity lymphedema treated with liposuction, researchers found that patients with early-stage disease demonstrated higher liposuction volumes, lower bleeding rates, and better volume improvement compared with those with advanced disease (P<0.05), indicating that disease duration directly impacts surgical efficacy (30). A retrospective analysis explicitly identified disease duration as a predictive factor for complete decongestive therapy efficacy, corroborating our finding of a negative correlation between duration and surgical outcomes (31).

Severity grade (based on preoperative limb volume excess) reflects the cumulative anatomical burden of disease. Our patients were classified as ISL stage II-III, representing the non-pitting edema phase. Within this homogeneous population, preoperative volume excess percentage further distinguished the degree of anatomical severity. In a study of post-traumatic lymphedema treated with LVA, Giunta et al. (32) reported that patients with preoperative limb volume excess of 8.68% achieved reduction to 0.12% at 12 months (98.54% reduction), whereas patients with preoperative volume excess of 13.35% achieved only 7.37% at 13 months (44.7% reduction). Although this study did not focus on BCRL, the pattern it revealed—smaller preoperative volume excess correlating with greater postoperative reduction rates—aligns with our finding that disease severity impacts long-term surgical outcomes. Furthermore, this volume-based stratification enables identification of high-risk patients who are unlikely to achieve satisfactory volume reduction even within the homogeneous non-pitting edema population, addressing the limitation of traditional staging systems in discriminating prognosis within the same pathological stage.

We acknowledge that using postoperative excess volume at day 7 to predict 6-month volume outcome introduces statistical dependency, as both reflect lymphatic drainage function. However, this represents a prospective time-series prediction rather than circular reasoning: postoperative excess volume at day 7 captures early anastomosis patency during the critical endothelial healing window (day 3-14), whereas 6-month outcome reflects long-term tissue remodeling and fibrosis stabilization. These are biologically distinct phases, with the former mechanistically determining the latter.

Crucially, sensitivity analysis demonstrated that even without postoperative excess volume, the remaining two variables (disease duration and severity grade) achieved substantial discrimination (AUC 0.726), confirming that these baseline characteristics provide independent prognostic information not redundant with early volume measurements. The 16.5% performance drop (P = 0.041) quantifies the unique contribution of early volume data, while AUC staying above 0.7 indicates the model still works using other factors alone, not just relying on early volume data.

The divergent behaviors of tree-based models versus linear SVM in this study reflect fundamental differences in statistical learning theory, particularly salient in our small-sample setting (n=211 training, p=3 predictors). Random Forest exhibited severe overfitting (training AUC 1.000 vs. validation 0.804), while linear SVM demonstrated stable generalization (training AUC 0.854 vs. validation 0.891).

This divergence reflects the fundamental bias-variance tradeoff in statistical learning theory, particularly salient in small-sample settings (n=211, p=3). Tree-based models possess high capacity to memorize training patterns. With limited samples (n/p ≈ 70), RF achieved apparent perfect discrimination (AUC 1.000), indicating that individual trees became overly complex and the ensemble effectively averaged memorized noise rather than generalizable signals—a phenomenon exacerbated when the number of predictors is small relative to sample size. This aligns with Breiman’s theory that random forest generalization depends on the correlation between trees (ρ̄) and individual tree strength (s)—when trees are too strong (s→1), the ensemble loses generalization capability (33).

Conversely, linear SVM employs structural risk minimization (SRM) rather than empirical risk minimization (34). By maximizing the geometric margin between classes, linear SVM imposes implicit regularization that constrains the hypothesis space, preventing overfitting. This explains the stable performance across training and validation sets, as the model prioritizes generalizable linear separation. This finding supports Vapnik’s principle that controlling model complexity via margin maximization yields better generalization than minimizing empirical error in small-sample settings (34).

Notably, this represents a deviation from typical performance hierarchies in large-scale machine learning, where ensemble trees often outperform linear models. In our clinical setting with limited samples and few robust predictors, linear SVM’s constrained complexity proved advantageous, suggesting that algorithm selection should consider dataset characteristics (n/p ratio) rather than presuming superior performance of complex models.

Currently, prediction models for long-term outcomes following lymphedema treatment are relatively scarce. The SVM model constructed in this study addresses this gap, demonstrating excellent discriminative ability in the validation set (AUC = 0.891). The model’s clinical utility is reflected in multidimensional performance metrics. The sensitivity of 90.8% facilitates accurate identification of most patients who will achieve satisfactory outcomes; the positive predictive value of 86.8% indicates that when the model predicts satisfactory outcome, the probability of actual favorable recovery is extremely high, supporting the formulation of routine rehabilitation protocols for these patients. Conversely, the specificity of 62.5% and negative predictive value of 71.4% suggest that when the model predicts residual swelling, the actual probability of residual swelling exceeds 70%; such patients should receive intensified intervention to avoid missing optimal treatment opportunities.

Compared with traditional predictive models, machine learning models often face implementation barriers in clinical decision-making due to their “black-box” characteristics. This study employed SHAP analysis to achieve individualized interpretation of SVM predictions (25). To facilitate clinical application of this predictive tool, we developed a user-friendly web-based application. This tool allows clinicians to input three characteristics—postoperative excess volume, disease duration, and severity grade—through a parameter panel to obtain immediate probability predictions for satisfactory outcome versus residual swelling. For high-risk patients identified by the model, closer monitoring may be warranted. The current model supports early risk stratification and provides a reference for future standardized rehabilitation studies.

This study has several limitations. First, as a single-center retrospective study of predominantly severe cases (ISL stage II–III), selection bias limits generalizability; ISL stage I patients undergo conservative rather than surgical treatment, restricting the model to advanced lymphedema indicated for combined SAL-LVA. Second, the Chinese cohort reflects ethnic and geographic homogeneity. Third, while bootstrap optimism correction (B = 1,000) confirmed robust internal validity (optimism-corrected AUC 0.863), external validation is lacking; we are disseminating the deep LVA technique to partner institutions to enable future multicenter validation. Fourth, as a retrospective study, patient-reported outcomes and garment dependence were not systematically collected, limiting assessment of patient-centered outcomes beyond volume metrics. Pending external validation, this model provides risk stratification for comparable specialized surgical populations only. .

## 5 Conclusion

This study developed and validated an SVM-based predictive model for six-month outcomes following combined surgery for breast cancer-related lymphedema. Utilizing three readily available indicators—postoperative excess limb volume, disease duration, and severity grade—the model effectively predicts therapeutic efficacy (AUC = 0.891). Integrated with SHAP analysis for interpretability and deployed as a web-based tool, this model enables clinicians to stratify patients by risk at discharge, thereby identifying those requiring closer monitoring and provides a reference for future protocolized rehabilitation research.

## Data Availability

The raw data supporting the conclusions of this article will be made available by the authors, without undue reservation.

## Supplementary material

Supplementary Table 1 Sensitivity Analysis: Performance of Reduced Models Excluding Postoperative Excess Volume.
