# Supplementary material for: Development and validation of a machine learning-based predictive model for early outcomes following combined suction-assisted lipectomy and lymphovenous anastomosis in breast cancer-related lymphedema: a retrospective cohort study
Source: Front Oncol. 2026 May 7;16:1828402. doi: 10.3389/fonc.2026.1828402 (PMC13189913; doi:10.3389/fonc.2026.1828402)
Supplement: Supplementary Table 1 — Sensitivity Analysis: Performance of Reduced Models Excluding Postoperative Excess Volume. [file Table1.docx]

Supplementary Table S1. Sensitivity Analysis: Performance of Reduced Models Excluding Postoperative Excess Volume

| Model | Variables | AUC (95% CI) | Sensitivity | Specificity | PPV | NPV |
| --- | --- | --- | --- | --- | --- | --- |
| SVM (Full) | 3 | 0.891 (0.810–0.950) | 90.80% | 62.50% | 86.80% | 71.40% |
| LightGBM (Reduced) | 2 | 0.726 (0.595–0.831) | 87.70% | 20.80% | 75.00% | 40.00% |

Footnote: DeLong test comparing full vs. reduced model: Z = 2.04, P = 0.041.
